# Identification and characterization of a novel extracellular polyhydroxyalkanoate depolymerase in the complete genome sequence of *Undibacterium* sp. KW1 and YM2 strains

**DOI:** 10.1371/journal.pone.0232698

**Published:** 2020-05-05

**Authors:** Tomohiro Morohoshi, Taishiro Oi, Tomohiro Suzuki, Shunsuke Sato

**Affiliations:** 1 Department of Material and Environmental Chemistry, Graduate School of Engineering, Utsunomiya University, Utsunomiya, Tochigi, Japan; 2 Center for Bioscience Research and Education, Utsunomiya University, Utsunomiya, Tochigi, Japan; 3 Biotechnology Laboratories, Pharma & Supplemental Nutrition Solutions Vehicle, Kaneka Corporation, Takasago, Hyogo, Japan; North Eastern Regional Institute of Science and Technology, INDIA

## Abstract

Polyhydroxyalkanoate (PHA) is a biodegradable polymer that is synthesized by a wide range of microorganisms. One of the derivatives of PHA, poly(3-hydroxybutyrate-*co*-3-hydroxyhexanoate) (PHBH) has flexible material properties and low melting temperature. We have previously demonstrated that PHBH is degradable in a freshwater environment via the formation of biofilm on the surface of the PHBH film. *Undibacterium* sp. KW1 and YM2, which were isolated from the biofilm present on the PHBH film in the freshwater sample, were shown to have PHBH-degrading activity. In this study, the complete genome sequence of KW1 and YM2 revealed that the extracellular PHA depolymerase gene, designated as *phaZ*_*UD*_, was present in their chromosomes. Sequence analysis revealed that PhaZ_UD_ contained four domains: a signal peptide, catalytic domain, linker domain, and substrate-binding domain. *Escherichia coli* harboring a PhaZ_UD_-expressing plasmid showed PHBH-degrading activity in LB medium containing 1 wt% PHBH powder. The recombinant His-tagged PhaZ_UD_ from KW1 and YM2 was purified from the culture supernatant and shown to have PHBH-degrading activity at the optimum temperature of 35 and 40°C, respectively. When the degradation product in the PHBH solution was treated with PhaZ_UD_ and assayed by LC-TOF-MS, we detected various oligomer structures, but no more than pentamers, which consist of 3-hydroxybutyrate and 3-hydroxyhexanoate. These results demonstrated that PhaZ_UD_ may have an endo-type extracellular PHA depolymerase activity.

## Introduction

In modern society, plastic pollution is a serious problem with serious effects on the environment and wildlife [1]. Especially, pollution by smaller pieces of plastic, called microplastic, has become a serious problem [2]. Microplastics are defined as particles less than 5 mm in size, which are generated from the plastic debris fragmented by UV radiation in the aquatic environment [2]. The use of biodegradable plastics is one of the solutions to prevent the generation of microplastics in the aquatic environment. Biodegradable plastics can be broken down by various bacteria, taken as a carbon source, and finally converted into CO_2_ [3]. Many kinds of oil- and bio-based biodegradable plastics have been developed and used to solve this serious environmental problem [3]. One of them, polyhydroxyalkanoate (PHA), is synthesized by a wide range of bacteria from renewable resources such as sugars, plant oils, glycerol, and others [4, 5]. It has been demonstrated that PHA is degradable in natural environments by PHA-degrading bacteria, which produce the extracellular PHA-degrading enzyme under both anaerobic and aerobic conditions [6]. For instance, poly(3-hydroxybutyrate) (PHB), one of the representative PHA derivatives, can be degraded by PHA depolymerase, which catalyzes the ester hydrolysis of PHB and converts it to 3-hydroxybutyrate (3HB) monomers or oligomers [7].

Poly(3-hydroxybutyrate-*co*-3-hydroxyhexanoate) (PHBH) is a random copolymer of 3HB and 3-hydroxyhexanoate (3HH) and has flexible material properties and a low melting temperature, which is advantageous due to lower temperatures needed for pyrolysis [8]. We have previously demonstrated that the PHBH film is degradable in both seawater and freshwater environments [8, 9]. In freshwater environments, PHBH-degrading bacteria isolated from the biofilm formed on the surface of the PHBH film were assigned to the genus *Acidovorax*, *Undibacterium*, and *Chitinimonas* [9]. Notably, the analysis of the bacterial community in the biofilms formed on the PHBH films showed that the genera *Acidovorax* and *Undibacterium* were predominant. Although the genus *Acidovorax* has been previously shown to be PHA-degrading bacteria [10, 11], there is no report of the genus *Undibacterium* as having this activity. Some bacterial strains, which are proposed as novel species within the genus *Undibacterium*, have been isolated from the freshwater samples or freshwater species [12, 13]. However, the physiological properties, genomic information, and genetic application of the genus *Undibacterium* have yet to be reported. In our previous study, we isolated two PHBH-degrading strains, *Undibacterium* sp., KW1 and YM2, from the Japanese lakes Kawaguchi-ko and Yamanaka-ko, respectively [9]. In this study, to elucidate the PHBH-degrading activity of the KW1 and YM2 strains, their complete genome was sequenced and a novel PHBH-degrading gene was identified. Moreover, we investigated the enzymatic activity of the purified PHBH-degrading enzyme.

## Materials and methods

### Bacterial strains, plasmids, compounds, and growth conditions

*Escherichia coli* was grown at 37°C in Luria-Bertani (LB) medium. *Undibacterium* sp. KW1 and YM2 were grown at 30°C in R2A medium (Difco, Tokyo, Japan). Solid bacterial media were prepared by adding agar to a final concentration of 1.5%. Antibiotics were added as required, at final concentrations of 100 and 20 μg/mL for ampicillin and chloramphenicol, respectively. The PHBH powder (KANEKA Biodegradable Polymer^™^ X131A: 3HHx = 6 mol%) was obtained from the Kaneka Corporation, Hyogo, Japan.

### Genome sequencing

The genomic DNA of the KW1 and YM2 strains was extracted using the DNeasy Blood & Tissue Kit (Qiagen, Tokyo, Japan). Genome sequencing was performed on the PacBio RSII platform (Pacific Biosciences, Menlo Park, CA) using libraries prepared with the SMRTbell Template Prep Kit 1.0 (Pacific Biosciences) by Macrogen Japan Corp. (Kyoto, Japan). The sequencing reads were assembled using the Canu version 1.6 software [14]. Prediction of putative coding sequences and gene annotations was performed using the Microbial Genome Annotation Pipeline (MiGAP) from the DNA Data Bank of Japan (DDBJ). Protein-coding sequences were predicted using a combination of MetaGeneAnnotator [15], RNAmmer [16], tRNAScan [17], and BLAST [18].

### Cloning of the phaZ gene from the *Undibacterium* strains

The *phaZ* genes from the KW1 and YM2 strains were amplified by PCR with KOD FX Neo DNA polymerase (Toyobo, Osaka, Japan) and the following primer sets: 5ʹ-TCTGGATCCCCAGGAATACGGACGTATGTCCACCC-3ʹ and 5ʹ-TCTAAGCTTCAGTAAAGGCTCAGGAGCAGACACGC-3ʹ for KW1, and 5ʹ-TCTGGATCCGGAATACGGAAGTATGTCCACCCTGG-3ʹ and 5ʹ-TCTAAGCTTCTGACTGCGCCTTATCAGATTCAGGG-3ʹ for YM2. The PCR was performed using the following cycling parameters: 98°C for 10 s and 68°C for 1 min for 30 cycles. The PCR fragments were digested with the *Bam*HI and *Hin*dIII (underlined) enzymes and inserted into the same restriction sites of the low copy vector pSTV28 (Takara Bio, Shiga, Japan). The constructed plasmids were transformed into *E*. *coli* DH5α cells and used for the PHBH degradation assay. The PHBH powder was mixed with LB medium at a concentration of 1 mg/mL and sonicated to obtain better dispersion. After being autoclaved, 10 mL of agar medium were poured into 60 mm plastic Petri dishes. *E*. *coli* cells harboring the *phaZ*-expressing plasmid were streaked onto the center of PHBH-containing LB plates. After incubation for one week at 30°C, the development of clear zones around the colonies was considered as degradation of PHBH.

### Expression and purification of His-tagged PhaZ

For the construction of the expression plasmid for His-tagged PhaZ from the KW1 and YM2 strains, the *phaZ* genes were amplified using the KOD FX Neo DNA polymerase and following primer sets: 5ʹ-TCTCATATGAATCATATCAAGACCATGTTGCGG-3ʹ and 5ʹ-TCTGGATCCTCAATGATGATGATGATGATGAGGGCAATTACCAATGATG-3ʹ for KW1, and 5ʹ-TCTCATATGAACAAGATCAAGATTATCTTGCAG-3ʹ and 5ʹ-TCTGGATCCTCAATGATGATGATGATGATGGGGGCAATTACCAATGATG-3ʹ for YM2. The PCR was performed using the following cycling parameters: 98°C for 10 s and 68°C for 1 min for 30 cycles. The pGEX28 plasmid, which is based on the low-copy vector pSTV28, was constructed for the expression of His-tagged PhaZ under the *tac* promoter in this study (S1 Fig). The PCR products were digested by the *Nde*I and *Bam*HI (underlined) enzymes and inserted into the same restriction sites on the pGEX28 plasmid to construct the pGEX28-KW1 (for PhaZ from KW1) and pGEX28-YM2 (for PhaZ from YM2) plasmid, respectively.

For the expression and purification of His-tagged PhaZ, a colony of *E*. *coli* BL21(DE3) harboring the pGEX28-KW1 or pGEX28-YM2 plasmid was directly inoculated into 100 mL of LB medium containing chloramphenicol. Cells were cultivated for approximately 8 h at 30°C until they reached the late exponential growth phase. Then, isopropyl-β-d-thiogalactoside (IPTG) was added at a final concentration of 500 μM. After incubation for 12 h at 30°C, the cell-free supernatant was prepared by centrifugation. Protein purification was performed using the ÄKTA start system (GE Healthcare, Tokyo, Japan). The filtrated cell-free supernatant was loaded onto a HisTrap affinity chromatography column (GE Healthcare) equilibrated with a loading buffer (20 mM sodium phosphate buffer, 500 mM NaCl, pH 7.4) containing 20 mM imidazole and eluted with the loading buffer containing 500 mM imidazole after washing off unbound bacterial proteins. The expression and purity of the His-tagged PhaZ were confirmed by SDS-PAGE.

### Assay of PHBH-degrading activity

To evaluate the PHBH-degrading activity of the purified PhaZ protein, a PHBH suspension was prepared, with a final concentration of 1 mg/mL in 10 mM Tris-HCl buffer (pH 8.0) and 1 mM CaCO_3_, and sonicated to obtain a better dispersion. To estimate the optimal temperature for the PHBH-degrading activity of PhaZ, 30 μL of the purified PhaZ solution was mixed with 270 μL of the PHBH solution. After incubation at 20 to 55°C for 1 h, 700 μL of distilled water was added. The PHBH-degrading activity was evaluated by measuring the decrease in turbidity at 600 nm and the maximum value was set to 100%. The experiment was repeated at least three times.

### LC-TOF-MS analysis of PHBH degradation products produced by PhaZ

A total of 500 μL of the purified PhaZ solution was mixed with 4.5 mL of the 5 mg/mL PHBH solution and statically incubated at 30°C until the turbidity disappeared. The chemical composition of the PHBH degradation products was analyzed by LC-TOF-MS. The PHBH degradation products were analyzed in negative ionization mode using a NexeraX2 (Shimadzu, Kyoto, Japan) coupled with a maXis4G mass spectrometer (Bruker Daltonics, Bremen, Germany) with an ACQUITY UPLC HSS T3 column (1.8 μm, 2.1 × 150 mm; Nihon Waters K.K., Tokyo, Japan) under the following conditions: ionization method; APCI method, column oven; 50°C, mass range; 50–1550 *m*/*z*. The mobile phase was 0.1% formic acid and an acetonitrile gradient and the flow rate was 0.5 mL/min.

### Nucleotide sequence accession number

The complete genome of *Undibacterium* sp. KW1 was deposited in the DDBJ/ENA/GenBank databases under the accession numbers AP018439 (chromosome) and AP018440 (plasmid pUKW01). The complete genome of *Undibacterium* sp. YM2 was deposited in the DDBJ/ENA/GenBank databases under the accession numbers AP018441 (chromosome) and AP018442 (plasmid pUYM01).

## Results and discussions

### The complete genome sequence revealed the presence of the PHA-depolymerase gene in *Undibacterium* strains

To identify the genes involved in the degradation of PHBH, we obtained the complete genome sequences of the KW1 and YM2 strains using the PacBio RSII platform. For the KW1 strain, we obtained 105,851 reads with an average length of 10,942 bp, which sequenced approximately 1.16 Gbp. After assembly, the genome of the KW1 strain included a single chromosome and one plasmid. The complete circular chromosome and the endogenous plasmid pUKW01 were 6,557,365 and 135,601 bp in size, respectively. For the YM2 strain, 145,259 reads with an average length of 10,896 bp were produced, which sequenced approximately 1.58 Gbp. After assembly, the genome of the YM2 strain also included a single chromosome and one plasmid. The complete circular chromosome and the endogenous plasmid pUYM01 were 6,484,812 and 175,363 bp in size, respectively. The complete genome of one genus *Undibacterium* strain, *Undibacterium parvum* DSM 23061, has been previously sequenced (accession number CP034464). The complete genome of DSM 23061 was shorter than that of KW1 and YM2 and did not contain the endogenous plasmid. The general features of the complete genome of the KW1 and YM2 strains are shown in Table 1.

**Table 1:** The general features of the genome of *Undibacterium* sp. KW1 and YM2, and *U*. *parvum* DSM 23061.

Species	*Undibacterium* sp.				*U*. *parvum*
Strain	KW1		YM2		DSM 23061
Replicon	Chromosome	Plasmid	Chromosome	Plasmid	Chromosome
Length (bp)	6557365	135601	6484812	175363	4910324
GC content (%)	51.0	50.3	51.2	50.5	50.2
CDSs	5890	90	5907	116	4143
rRNA operons	4	0	4	0	6
tRNAs	89	0	93	0	62

Subsequently, the presence of a PHA depolymerase gene homolog was investigated in the complete genomes of the KW1 and YM2 strains. We found that the amino acid sequences of UNDKW_2839 and UNDKW_4104 from KW1, and UNDYM_2916 and UNDYM_4081 from YM2 showed high similarities to the known PHA depolymerase. Generally, crystalline PHA-based polymers, such as the PHBH film, can be degraded by extracellular PHA depolymerase but not the intracellular version of this enzyme [10]. The results of the SignalP analysis [19] showed that the UNDKW_4104 sequence from the KW1 strain and the UNDYM_4081 sequence from the YM2 strain were predicted to be extracellular proteins with an N-terminal signal peptide (SP) of 26 amino acid residues. Therefore, UNDKW_4104 and UNDYM_4081 were identified as the extracellular PHA depolymerase gene involved in PHBH degradation and designed as the *phaZ*_*UD*_ gene. On the other hand, since the UNDKW_2839 sequence from the KW1 strain and the UNDYM_2916 sequence from the YM2 strain did not contain N-terminal signal peptides, it is possible that these proteins may act as intracellular PHA depolymerases.

### In silico characterization of the PhaZ_UD_ homolog from the family *Oxalobacteraceae*

**Fig 1:**
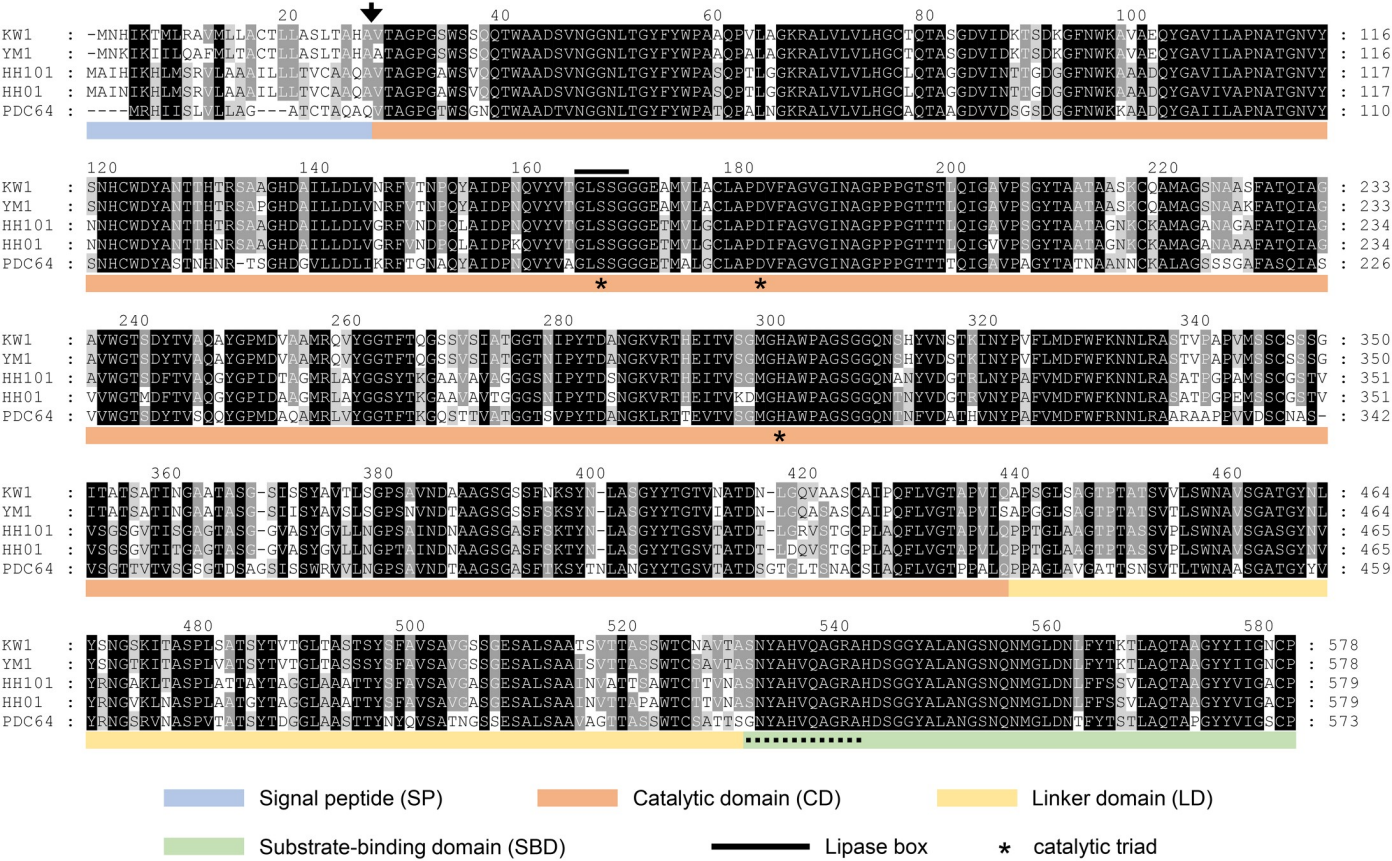
Amino acid sequence and alignment of PhaZ_UD_ and PhaZ homologs from the family *Oxalobacteraceae*. The putative SP, CD, LD, and SBD were marked in blue, orange, yellow, and green, respectively. The predicted cleavage site of the N-terminal signal peptide was labeled by an arrow. The putative catalytic triad was marked by asterisks. The conserved lipase box sequence was shown by an overline. The conserved motif sequence of SBD domain was shown by a dotted underline. PhaZ_UD_ were compared with extracellular PhaZ homologs from the family *Oxalobacteraceae*, *Duganella* sp. HH101 (UniProt accession no. A0A1E7WFX8), *Janthinobacterium* sp. HH01 (L9PFG0), and *Massilia* sp. PDC64 (A0A1G7ANH7).

The known PHA depolymerases are composed of a catalytic domain (CD) at the N terminus, substrate-binding domain (SBD) at the C terminus, and linker domain (LD) connecting the two previously mentioned domains [20]. Sequence analysis revealed that the putative PhaZ_UD_ contained four domains, SP, CD, LD, and SBD (Fig 1). PHA depolymerases have a catalytic triad as the active site and the catalytic serine is embedded in a GXSXG sequence motif known as a lipase box [7]. The catalytic triad Ser165, Asp240, and His299 was found in the PhaZ_UD_ protein and Ser165 was also one of the residues in the lipase box-like sequence (GLSSG) (Fig 1). In general PhaZ sequences, the LD domain showed a high degree of homology to the fibronectin type III domain [21]. PROSITE motif scan [22] revealed the presence of a fibronectin type III domain homolog in the PhaZ protein from the KW1 and YM2 strains. The conserved motif sequence (sxxxHxxAGRa) was observed in all SBDs in known PhaZ sequences [21]. The SBD-like sequences (SNYAHVQAGRA) were also found at the C-termini in PhaZ_UD_ (Fig 1). These results demonstrate that the features of previously reported PHA depolymerases were well-conserved in PhaZ_UD_.

The results of the BLAST search revealed that PhaZ_UD_ showed high similarity (over 80%) to a PhaZ homolog from bacteria belonging to the family *Oxalobacteraceae*, namely *Duganella* sp. HH101, *Janthinobacterium* sp. HH01, and *Massilia* sp. PDC64 (Fig 1). Similarly to PhaZ_UD_, the PhaZ homologs of the family *Oxalobacteraceae* also conserved the SP, catalytic triad (Ser, Asp, and His), lipase box-like sequence, and SBD motif sequence (Fig 1). To investigate the distribution of the PhaZ homolog in the family *Oxalobacteraceae*, we searched for the presence of the *phaZ* gene homolog in the complete genome of 24 strains belonging to this family in the NCBI GenBank database. The results of the BLAST search revealed that the *phaZ* gene homolog was present in the complete genome of some strains belonging to the genus *Massilia*, *Janthinobacterium*, and *Herbaspirillum*, but not in *Collimonas* and *Oxalobacter* (S2 Fig). Although the complete genome sequence of the *Undibacterium parvum* type strain DSM 23061 possesses the putative intracellular PHA depolymerase gene (EJN92_20730), the extracellular PHA depolymerase gene homolog, which contains an SP at its N-terminus, was not found in that genome. The length of the chromosome of the DSM 23061 strain (approximately 4.9 Mbp) was shorter than that of the KW1 and YM2 strains (approximately 6.5 Mbp) (Table 1). It is possible that the chromosome of the DSM 23061 strain was shortened due to the process of evolution and, therefore, lost the extracellular PHA depolymerase gene.

### The PhaZ_UD_ proteins have PHBH-degrading activity

As the KW1 and YM2 strains were isolated from a freshwater environment as PHBH-degrading bacteria, the PhaZ_UD_ proteins from the two strains were evaluated for their PHBH-degrading activities. Because intracellular expression of PhaZ_UD_ from the KW1 and YM2 strains in *E*. *coli* cell caused a growth defect in our experimental conditions, the *phaZ*_*UD*_ gene from the KW1 and YM2 strains was subcloned into the low-copy pSTV28 vector based on the p15A origin. *E*. *coli* harboring the PhaZ_UD_-expressing plasmid were checked for PHBH-degrading activity on LB medium containing 1 wt% PHBH powder. After incubation for one week at 30°C, the development of clear zones around the colonies was observed (S3 Fig). On the other hand, *E*. *coli* harboring the putative intracellular PHA depolymerase genes, UNDKW_2839 from the KW1 strain and UNDYM_2916 from the YM2 strain, did not show PHBH-degrading activity (S3 Fig). These results demonstrated that PhaZ_UD_ may function as an extracellular PHA depolymerase and be involved in the degradation of PHBH in the parent strains, *Undibacterium* sp. KW1 and YM2.

### Expression, purification, and characterization of PhaZ_UD_

**Fig 2:**
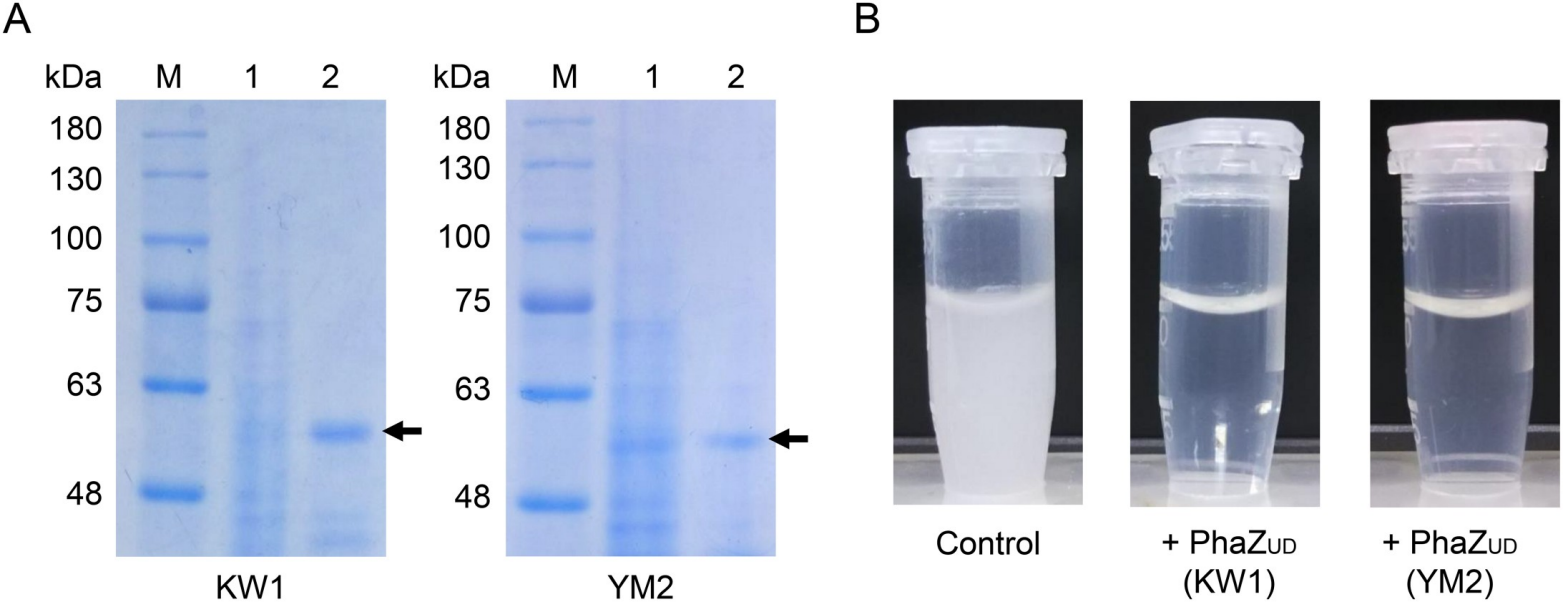
Purification and characterization of His-tagged PhaZ_UD_. (A) Purification of His-tagged PhaZ_UD_ from the culture supernatant of the KW1 and YM2 strains. Lane M, protein molecular weight marker (Nihon Genetics, Tokyo, Japan); lane 1, cell-free culture supernatant; and lane 2, purified His-tagged PhaZ_UD_ from KW1 or YM2. Samples were analyzed by SDS-PAGE in a 10% polyacrylamide gel. The approximately 56 kDa protein band of PhaZ_UD_ was indicated by an arrow. (B) The PHBH-degrading activity of His-tagged PhaZ_UD_. A total of 200 μL of purified PhaZ_UD_ sample was mixed with 800 μL of 5 mg/mL PHBH solution in a 1.5 mL microtube. After incubation for 4 h at 30°C, PHBH-degrading activity was observed as a drastic decrease in turbidity in the samples mixed with PhaZ_UD_.

To obtain the purified PhaZ_UD_ protein, the recombinant plasmid for expressing PhaZ_UD_, which was fused with a histidine tag at the C-terminus, was constructed and transformed into *E*. *coli* BL21(DE3) cells. Since the His-tagged PhaZ_UD_ preserved its SP sequence at N-terminal, the His-tagged PhaZ_UD_ were secreted and therefore purified from the culture supernatant. The results from the SDS-PAGE revealed that the overexpressed products were approximately 56 kDa in size, as expected (Fig 2A). To confirm whether the two PhaZ_UD_ proteins have PHBH-degrading activity, the purified His-tagged PhaZ_UD_ was mixed with PHBH solution and incubated at 30°C. After 4 h incubation, the turbidity of the PHBH solution treated with His-tagged PhaZ_UD_ was significantly decreased (Fig 2B). These results demonstrated that the PhaZ_UD_ proteins have PHBH-degrading activity and that the protein is secreted.

**Fig 3:**
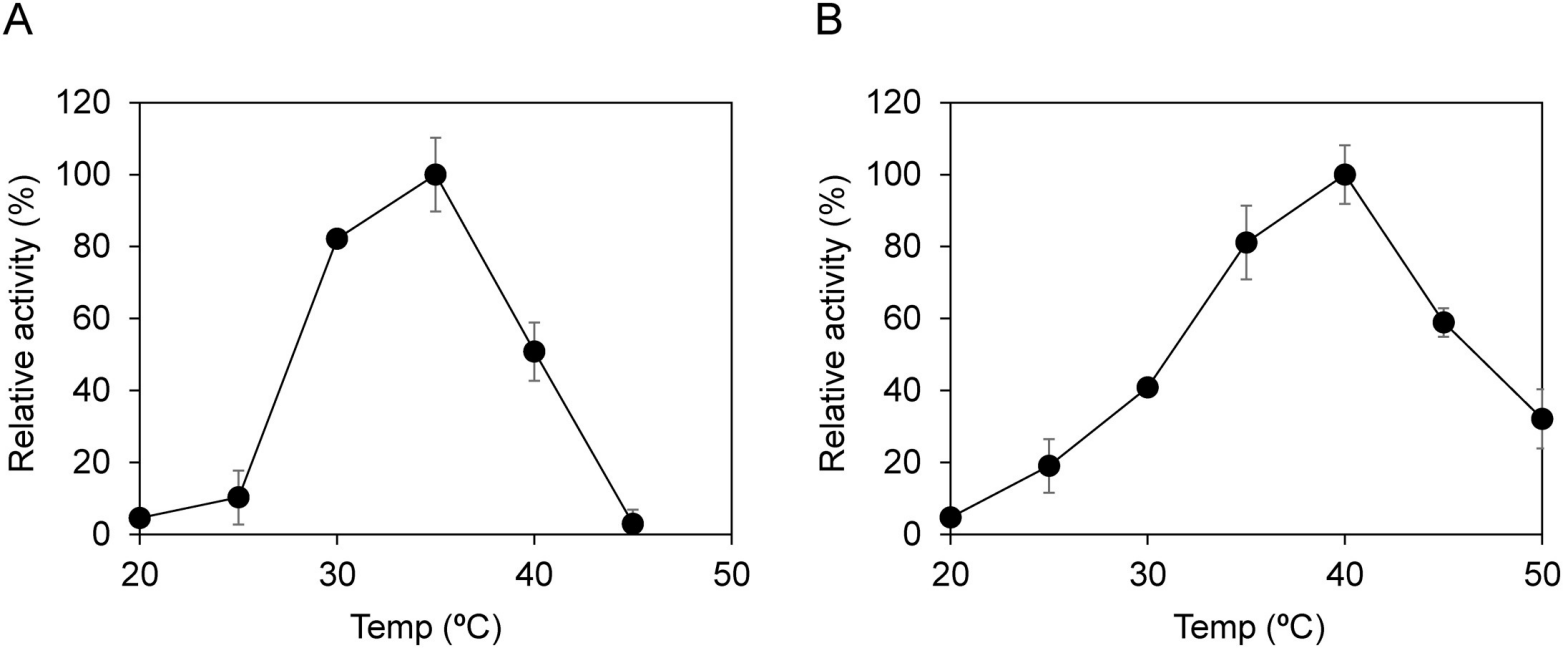
Assay for the optimal temperature of PhaZ_UD_. The purified PhaZ_UD_ solution from the KW1 (A) and YM2 (B) strains was mixed with 1 mg/mL PHBH solution and incubated at temperatures ranging from 20 to 50°C. After incubation for 1 h, PHBH-degrading activity was evaluated by measuring the decrease in turbidity at 600 nm. The maximum activity of each PHBH degradation was defined as 100%. The experiments were performed at least three times and error bars indicate standard deviations.

To estimate the optimal temperature for the PHBH-degrading activity of PhaZ_UD_, the PHBH powder was incubated with PhaZ_UD_ at various temperatures. His-tagged PhaZ_UD_ from the KW1 and YM2 strains displayed over 80% of its maximum activity between 30 and 40°C and the optimum temperatures of PhaZ_UD_ from the KW1 and YM2 strains were 35 and 40°C, respectively (Fig 3). On the other hand, the relative activity was greatly reduced at over 45°C. In a previous study, the enzymatic properties of the PHA depolymerase from *Alcaligenes faecalis* AE122 and *Acidovorax* sp. DP5, which belonged to the order *Burkholderiales*, were characterized [10, 23]. Since the optimum temperature for PHA-degrading activity of the proteins from *A*. *faecalis* AE122 and *Acidovorax* sp. DP5 were 55 and 40°C, respectively, the optimum temperature of PhaZ_UD_ was similar to that of *Acidovorax* sp. We have demonstrated that the genus *Acidovorax* and *Undibacterium* are the dominant PHBH-degrading bacteria in the biofilms formed on the PHBH film in freshwater samples [9]. Therefore, it was assumed that PHBH-degrading enzymes with similar properties were secreted by the *Undibacterium* and its related bacteria in the biofilm, which were then used to degrade the PHBH films.

### PhaZ_UD_ works as an endo-type PHA depolymerase

To elucidate the mechanism of PHBH degradation by PhaZ_UD_, we investigated the degradation products in the PHBH solution treated with PhaZ_UD_ from the KW1 strain by LC-TOF-MS. The His-tagged PhaZ_UD_-treated PHBH solution was used for LC-TOF-MS assay just before its turbidity completely disappeared. The detected PHBH monomers or oligomers, which was derived from PHBH degradation by PhaZ_UD_, were listed in Table 2. In this assay, oligomers with a molecular weight of 1,000 or more were not detected. The detected oligomers with a higher molecular weight were tetramers and pentamers, which consisted of two 3HB and 3HH, and two 3HB and three HH, respectively. The detected trimers contained three types of composition, which were 3HB-3HH-3HB, 3HH-3HB-3HH, and 3HH-3HH-3HB. The detected dimers contained 3HB-3HH and 3HB-3HB, but not 3HH-3HH. The detected monomers contained both 3HB and 3HH. Due to the fact that oligomers of various lengths were detected as the degradation product in the PHBH solution treated with PhaZ_UD_, it was suggested that PhaZ_UD_ may have at least an endo-type PHA depolymerase activity, and might have an exo-type PHA depolymerase and/or PHA oligomer hydrolase activity. We used a PHBH powder containing 3HH at a concentration of 6 mol% in this study. Although 3HB-3HB bonds are present at a higher ratio than 3HH-3HB and 3HH-3HH, all three structure of the detected trimers contained a 3HH unit and the 3HB-3HB-3HB trimer and 3HH-3HH dimer was not detected in the PhaZ_UD_-treated PHBH solution. These features suggested that PhaZ had a lower hydrolysis activity against PHBH oligomers that contained multiple 3HH units.

**Table 2:** Chemical formula, ionization mode, theoretical and measured *m*/*z* value, and retention time for detected metabolites.

Compound	Formula	Ionization mode	Measured *m*/*z*	Theoretical *m*/*z*	Retention time (min)
3HB	C_4_H_8_O_3_	[M-H]^-^	103.040443	103.040068	1.6
3HH	C_6_H_12_O_3_	[M-H]^-^	131.071072	131.071368	5.3
3HB-3HB	C_8_H_14_O_5_	[M-H]^+^	191.092459	191.091400	5.5
		[M-H]^-^	189.076816	189.076847	5.8
3HB-3HH	C_10_H_18_O_5_	[M-H]^+^	219.123039	219.122700	6.9
		[M-H]^-^	217.107740	217.108147	7.2
3HB-3HH-3HB	C_14_H_24_O_7_	[M-H]^+^	305.160259	305.159480	7.8
		[M-H]^-^	303.144080	303.144927	8.0
3HH-3HB-3HH	C_16_H_28_O_7_	[M-H]^+^	333.190861	333.190780	8.8
		[M-H]^-^	331.175475	331.176227	9.0
3HH-3HH-3HB	C_16_H_28_O_7_	[M-H]^+^	333.190748	333.190780	9.0
		[M-H]^-^	331.174739	331.174879	9.2
3HB×2, 3HH×2	C_20_H_34_O_9_	[M-H]^+^	419.228048	419.227559	9.4
		[M-H]^-^	417.212932	417.213006	9.6
3HB×2, 3HH×3	C_26_H_44_O_11_	[M-H]^+^	533.295312	533.295639	10.4

### Conclusions

In this study, we demonstrated that the genome sequences of the *Undibacterium* sp. KW1 and YM2 contain the *phaZ* gene homolog and that PhaZ_UD_ degrades PHBH by acting as an endo-type PHA depolymerase. The genus *Undibacterium* belongs to the family *Oxalobacteraceae*, which is a phylogenetically diverse aquatic bacterium family [24]. Although the family *Oxalobacteraceae* includes the genera *Oxalobacter*, *Collimonas*, *Massilia*, *Duganella*, *Janthinobacterium*, *Herbaspirillum*, and *Undibacterium* [24], it has not been reported that these genera have extracellular PHA-degrading activity and possess the *phaZ* gene homolog in their genome. The bacterial strains belonging to the genus *Undibacterium* has been isolated from the various aquatic environments. However, the role of genus *Undibacterium* in such environments remains unclear. We have previously demonstrated that the genus *Undibacterium* was predominant in the biofilm formed on the PHBH films in freshwater samples obtained from various places in Japan [9]. In the biofilm, it is possible that the genus *Undibacterium* produce extracellular PHA depolymerase, degrade PHBH films, and uptake PHBH oligomers with reduced molecular weight as a carbon source. Therefore, in aquatic environments where the genus *Undibacterium* might be ubiquitous, PHBH-based polymers may be an attractive choice, as this type of biodegradable might not remain as microplastic due to degradation.

## Supporting information

S1 Fig Vector map of pGEX28. (TIF)

S2 Fig Phylogenetic tree based on the complete sequences of chromosome of 24 strains belonged to the family *Oxalobacteraceae* in NCBI GenBank database. The name of bacterial strains, which have the *phaZ* gene homolog in their complete genome, was shown in blue.
(TIF)

S3 Fig PHBH-degrading activity of *E*. *coli* harboring pUC118-based plasmids. PHBH-degrading activity was detected on the LB agar plate containing 1 mg/mL PHBH powder after incubation for one week at 30°C. The development of clear zones around the colonies was evaluated as a degradation of PHBH.
(TIF)

S1 Table Raw data for Fig 3. (XLSX)

S1 Raw Images (PDF)
