# Supplementary figures and images for: Identification and characterization of a novel extracellular polyhydroxyalkanoate depolymerase in the complete genome sequence of Undibacterium sp. KW1 and YM2 strains
Source: PLoS One. 2020 May 5;15(5):e0232698. doi: 10.1371/journal.pone.0232698 (PMC7199957; doi:10.1371/journal.pone.0232698)

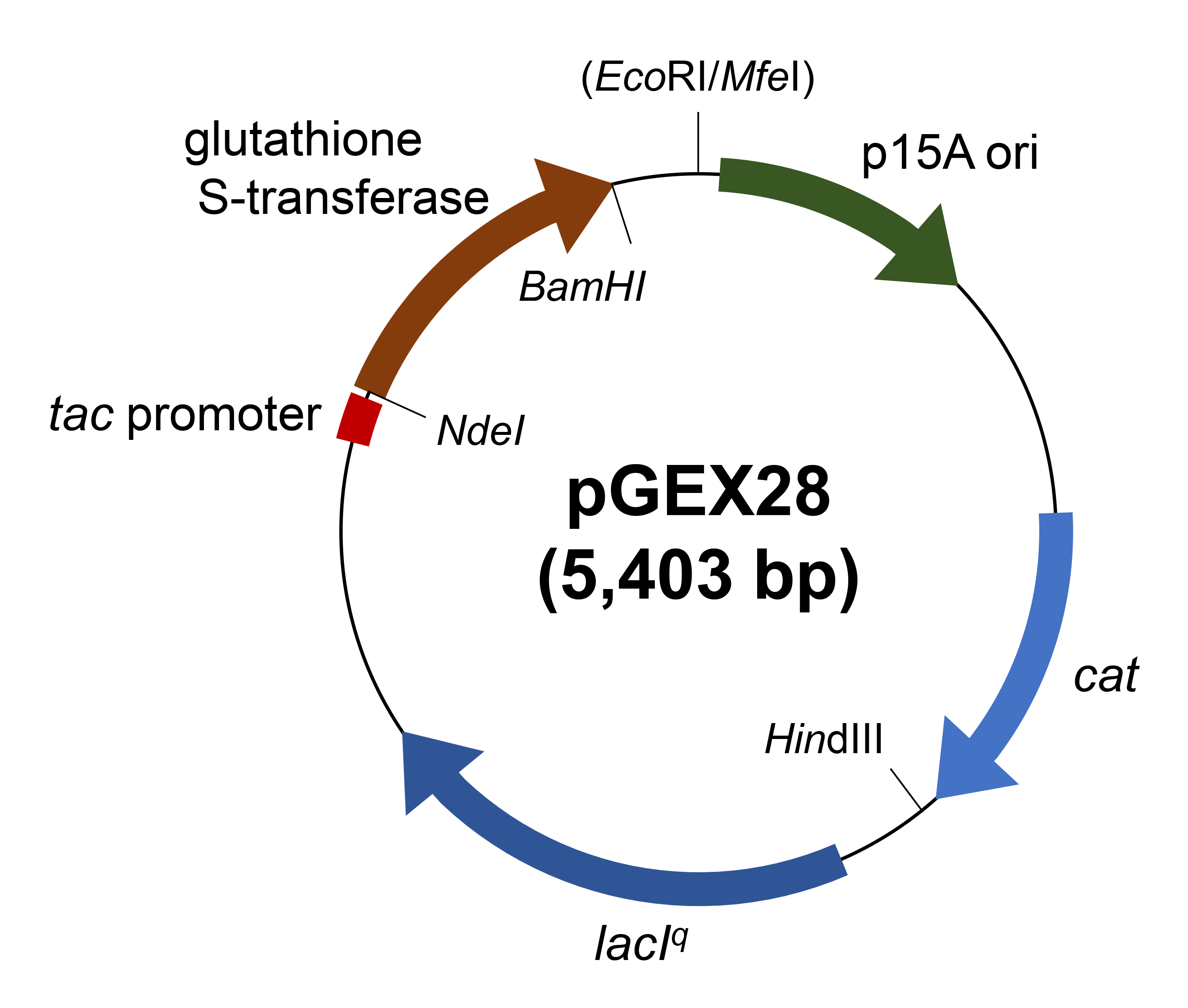

Supplement: S1 Fig — (TIF) [file pone.0232698.s001.tif]

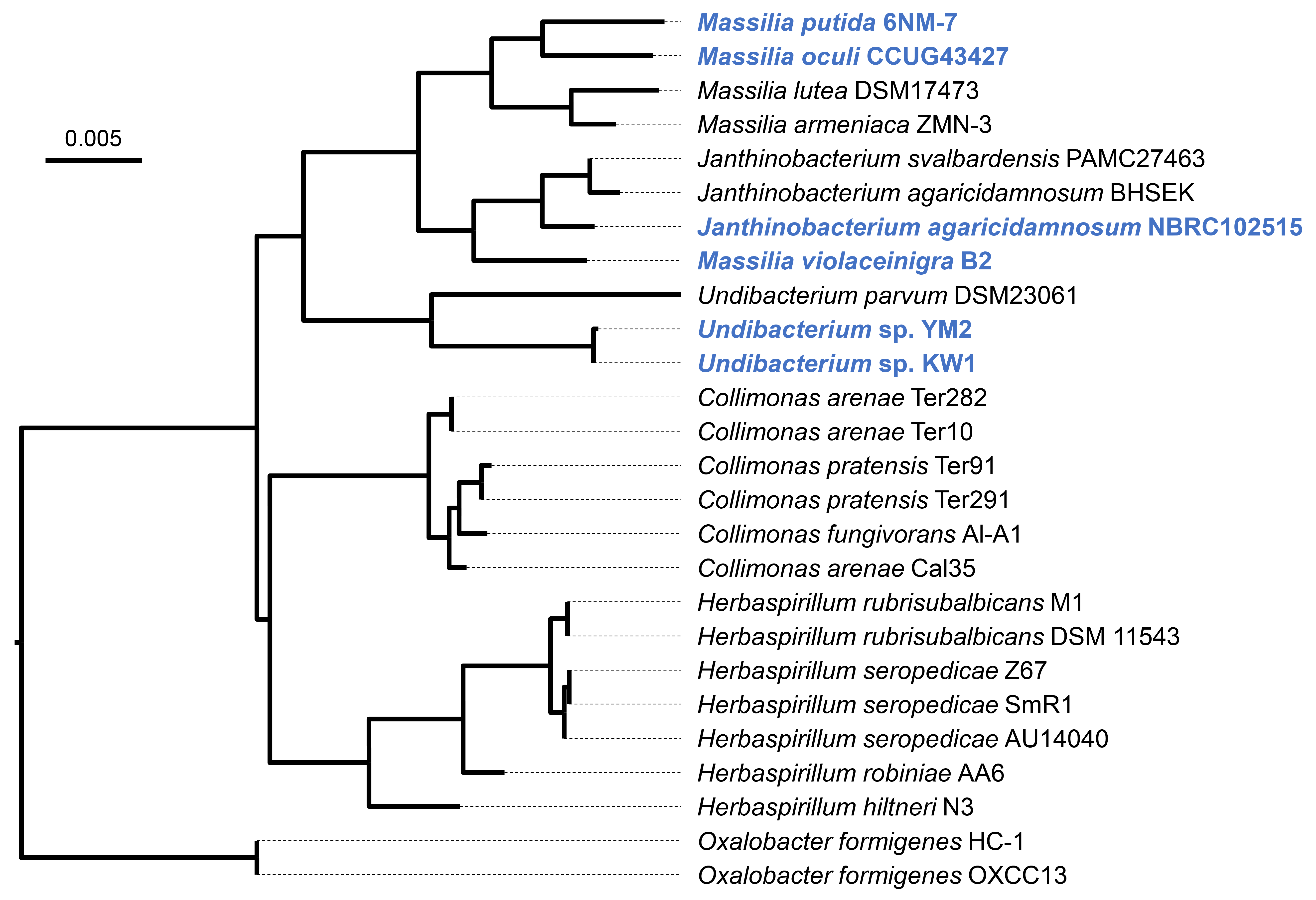

Supplement: S2 Fig — The name of bacterial strains, which have the phaZ gene homolog in their complete genome, was shown in blue. (TIF) [file pone.0232698.s002.tif]

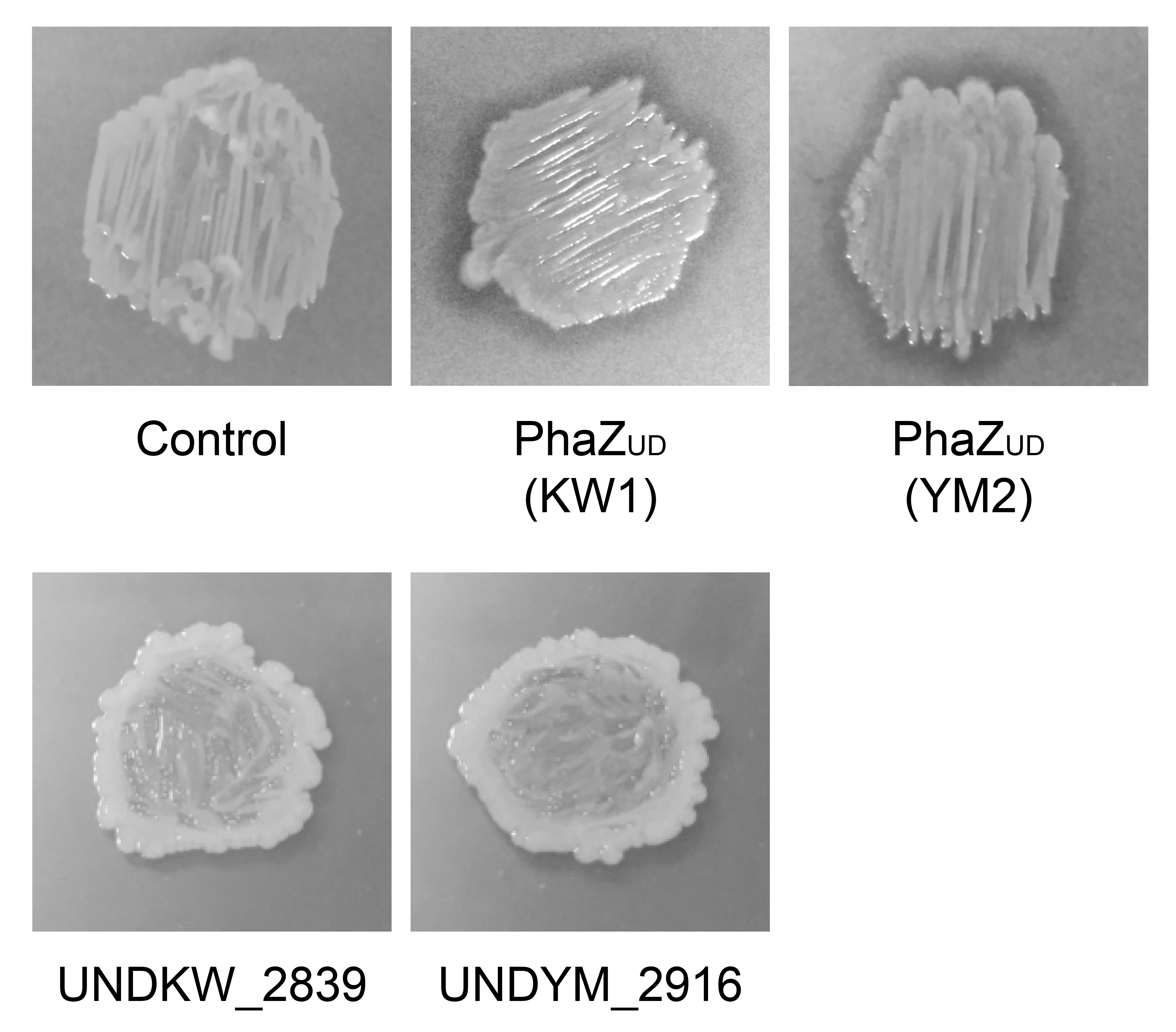

Supplement: S3 Fig — PHBH-degrading activity was detected on the LB agar plate containing 1 mg/mL PHBH powder after incubation for one week at 30°C. The development of clear zones around the colonies was evaluated as a degradation of PHBH. (TIF) [file pone.0232698.s003.tif]

Fig. 3A (KW1)

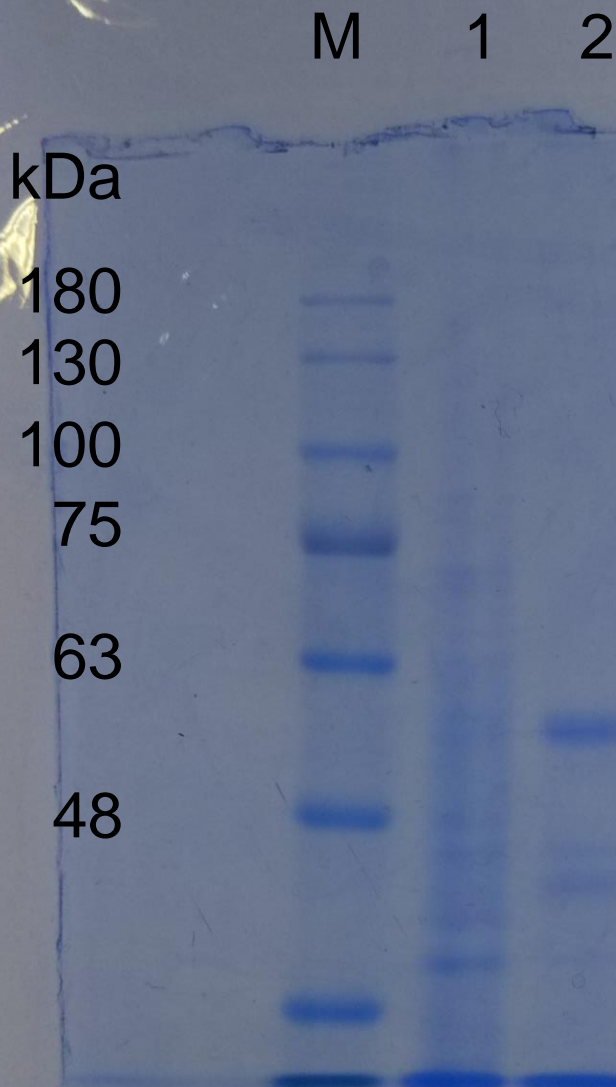

Fig. 3A  
(YM2)

kDa 180

130

100

75

63

48

M

1

2

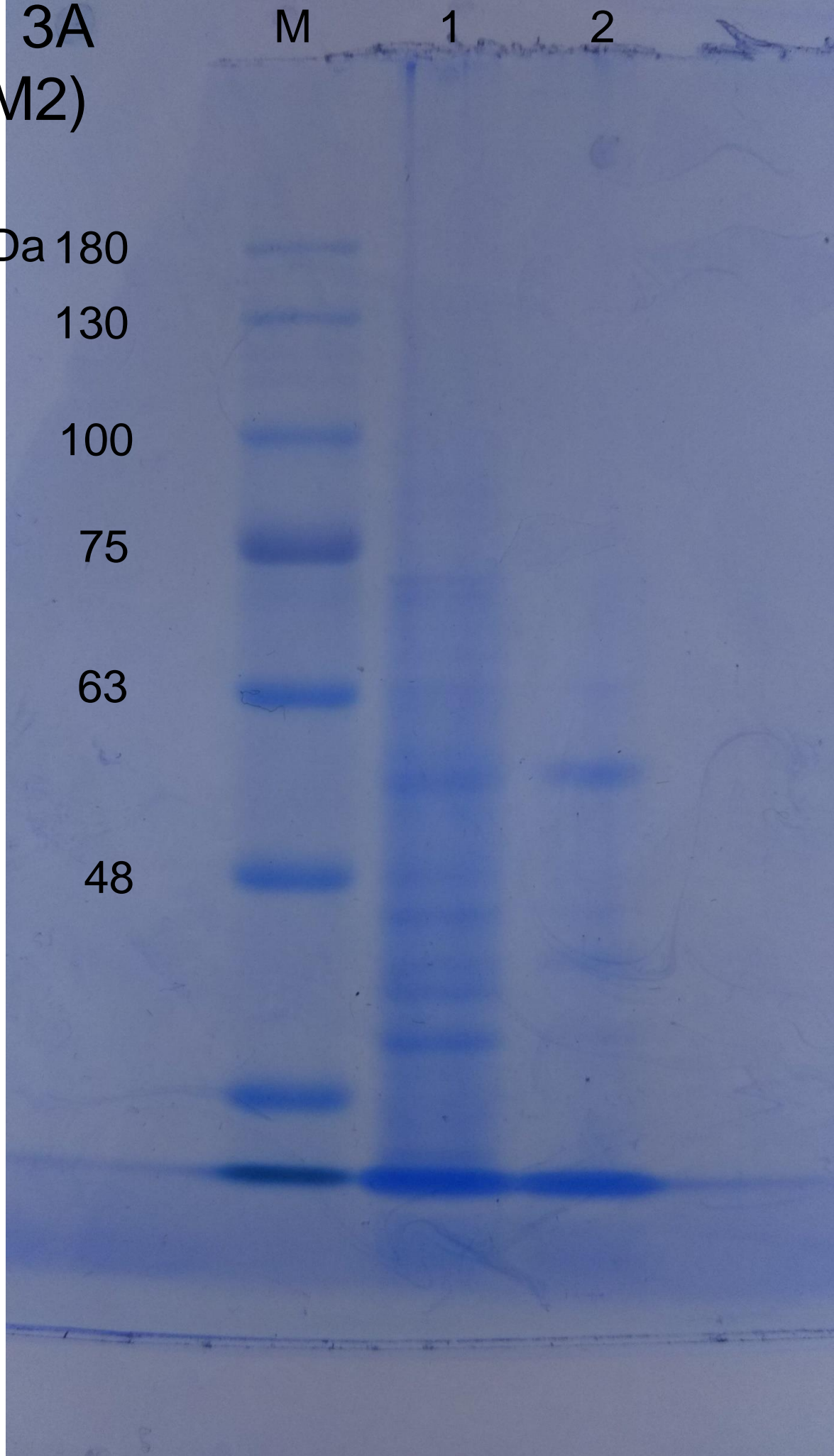

Supplement: S1 Raw Images — (PDF) [file pone.0232698.s005.pdf]
